# Acute, Recent and Past HEV Infection among Voluntary Blood Donors in China: A Systematic Review and Meta-Analysis

**DOI:** 10.1371/journal.pone.0161089

**Published:** 2016-09-06

**Authors:** Meiyu Wang, Ping Fu, Yonghua Yin, Miao He, Yu Liu

**Affiliations:** Institute of Blood Transfusion, Chinese Academy of Medical Sciences, HuaCai Road 26 Hao, Dong San Huan Road ErDuan, Chengdu, Sichuan, China; Centers for Disease Control and Prevention, UNITED STATES

## Abstract

**Introduction:**

Hepatitis E virus is one of new threats to blood safety which was usually considered to be transmitted via fecal-oral route. China is one of the hyperendemic regions where frequent outbreaks of hepatitis E are noted. However, the overall prevalence of HEV infection among mainland Chinese blood donors is not clear until now.

**Method:**

The peer-reviewed literatures reporting the prevalence of HEV in Chinese blood donors were identified by systematic searching of five electronic databases. The systematic review and meta-analysis were conducted in accordance with the Preferred Reporting Items for Systematic Reviews and Meta-Analyses statement issued in 2009. Data manipulation and statistical analyses were performed by Stata 12.0.

**Results:**

Fourteen eligible articles involving 22 independent studies were included. Pooled prevalence of HEV infection biomarkers (anti-HEV IgG, anti-HEV IgM, RNA and antigen) among mainland Chinese blood donors were 29.2%, 1.1%, 0.1% and 0.1%, respectively which were higher than the data reported in other countries. The analysis of HEV genotypes indicated that the most prevalent strains in Chinese blood donors were genotype 1 and 4.

**Conclusions:**

Mainland China is indicated with a relatively higher risk of transmission of hepatitis E through transfusion and the screening of blood donors for HEV RNA, especially in HEV-endemic areas, might reduce the potential risk of HEV infection via transfusion.

## Introduction

Hepatitis E virus (HEV) infection is the main cause of both epidemic and sporadic hepatitis E all over the world. In June 2014, the World Health Organization (WHO) report estimated 20 million hepatitis E infections were found each year with over three million acute cases of hepatitis E and 56,600 hepatitis E-related deaths[1]. China is one of the highly prevalent regions of HEV where hepatitis E outbreaks were reported several times. One of the biggest outbreaks occurred in Xinjiang in 1980s with a total of 119,280 individual infected[2].

Hepatitis E virus (HEV)is a non-enveloped RNA virus with a positive sense single-stranded genome of approximately 7.2 kb containing three open reading frames[3]. Genomic sequence analysis have classified HEV strains among humans into four genotypes and several subgenotypes[3,4].

Since the clinical symptoms of HEV infection are atypical usually, diagnosis of HEV infection is mainly based on the testing of HEV infection related biomarkers including HEV RNA, HEV antigen, anti-HEV IgG and anti-HEV IgM[5–7]. The incubation period, from HEV exposure to the onset of symptoms, ranges from 3 to 8 weeks generally[8]. After that, a sharp elevation of serum alanine transaminase (ALT) level coincides with the onset of symptoms in acute hepatitis E. HEV RNA can be detected in serum during the incubation period and early acute phase of the infection and the viremia can last for nearly up to 7 weeks after the onset of the symptom [8]. Generally, HEV antigens are detected before or along with HEV RNA[9,10]. Specific immune responses occur during the late incubation period, the titer of anti-HEV IgM increases rapidly and then wanes after several weeks[11]. Meanwhile, the titer of anti-HEV IgG continues to rise during the convalescent period and may persist in serum for a long time after the clearance of the virus[9].

HEV is primarily transmitted due to faecal contamination of drinking water, other routes such as food-borne transmission and vertical transmission have been documented as well[3,4]. Some evidences have suggested that HEV can be transmitted through blood transfusion and aroused increasing concerns about the blood safety in many countries. Cases of transfusion transmitted HEV have been reported in UK, Japan, Saudi Arabia, India and some other countries, while no such case has been reported in China so far[12–15]. Prevalence of HEV in Chinese blood donors has been reported by some articles. However, the multi-center large-scale data of this is scarce. This systematic review and meta-analysis was conducted to gain an overall insight into the prevalent characteristics of HEV infection in Chinese blood donors and to evaluate the risk of HEV infection for blood safety in China.

## Materials and Methods

This meta-analysis was conducted and reported according to the PRISMA (Preferred Reporting Items for Systematic Reviews and Meta-Analyses) Statement issued in 2015 strictly[16].

### Literature Search

Systematic searches were conducted in the major English and Chinese electronic databases (PubMed, Wiley Online Library Data, Science Direct, CNKI and Wanfang Data) for the literatures published from January 1999 to December 2014. To search and include as many related studies as possible, we used combinations of the next three groups of keywords: a) hepatitis E or HEV, b) blood donors or donation, c) China or Chinese. Every search was combined with the set operator “NO” and the terms of Taiwan, Macao or Hong Kong to exclude the studies which were not conducted in mainland China. A manual search of the reference lists of published articles was also performed.

### Selection Criteria and Data Abstraction

Inclusion criteria for identified articles were as follows: 1) full text articles in the electronic database mentioned above; 2) studies reported HEV infection in Chinese mainland blood donors; 3) studies were conducted among voluntary blood donors; 4) studies with clear sample size and exact number of HEV infection cases; 5) studies were conducted after 1999 (the year of implementation of Blood Donation Law).

Exclusion criteria were as follows: 1) studies conducted outside mainland China such as Hong Kong, Macao or Taiwan; 2) comments, reviews or conference abstracts; 3) studies performed before January 1999; 4) studies involving paid donors’ samples. Moreover, for publications based on the same sample origin, the ones with less information were excluded. The data were extracted by two experienced investigators independently from eligible studies. All discrepancies were settled by discussion to achieve consensus. The following information was extracted from the eligible studies: first author’s name, year of publication, year of sampling, study location, sample collection period, sample size, gender ratio, testing methods of different HEV biomarkers, ALT values and number of cases positive for anti-HEV IgG, HEV antigen anti-HEV IgM or HEV RNA respectively. The studies contain several sub-studies which performed in different areas were treated as several independent studies.

### Statistical Analysis

Data manipulation and statistical analysis was carried out using Stata 12.0 (Stata Corp LP, College Station, Texas, USA). The prevalence data were assumed to be the effective estimates. Sampling variance was represented by the standard error (ser) and calculated with *n* as the sample size and *p* as the positive rate. The prevalence of every marker was combined as a pooled prevalence for all studies and corresponding 95% confidence interval(IC) was determined based on random or fixed effects models, taking into account the possibility of heterogeneity between studies, which was tested using the Q test (p < 0.10 was considered indicative of statistically significant heterogeneity) and I^2^ test (values of 25%, 50%, and 75% were considered to represent low, medium, and high heterogeneity, respectively). Sub-group analysis on potential factors that associated with heterogeneity was performed, following by meta-regression on the period of sampling, sample size, gender ratio and the proportion of the cases with elevated ALT. The factor R^2^ more than 5% or p value smaller than 0.1 was considered to contribute to between-study heterogeneity significantly[17]. In addition, the publication bias was assessed by Egger’s tests, in which p < 0.05 represents statistically significant publication bias.

## Results

### Process of Studies Selection

**Fig 1:**
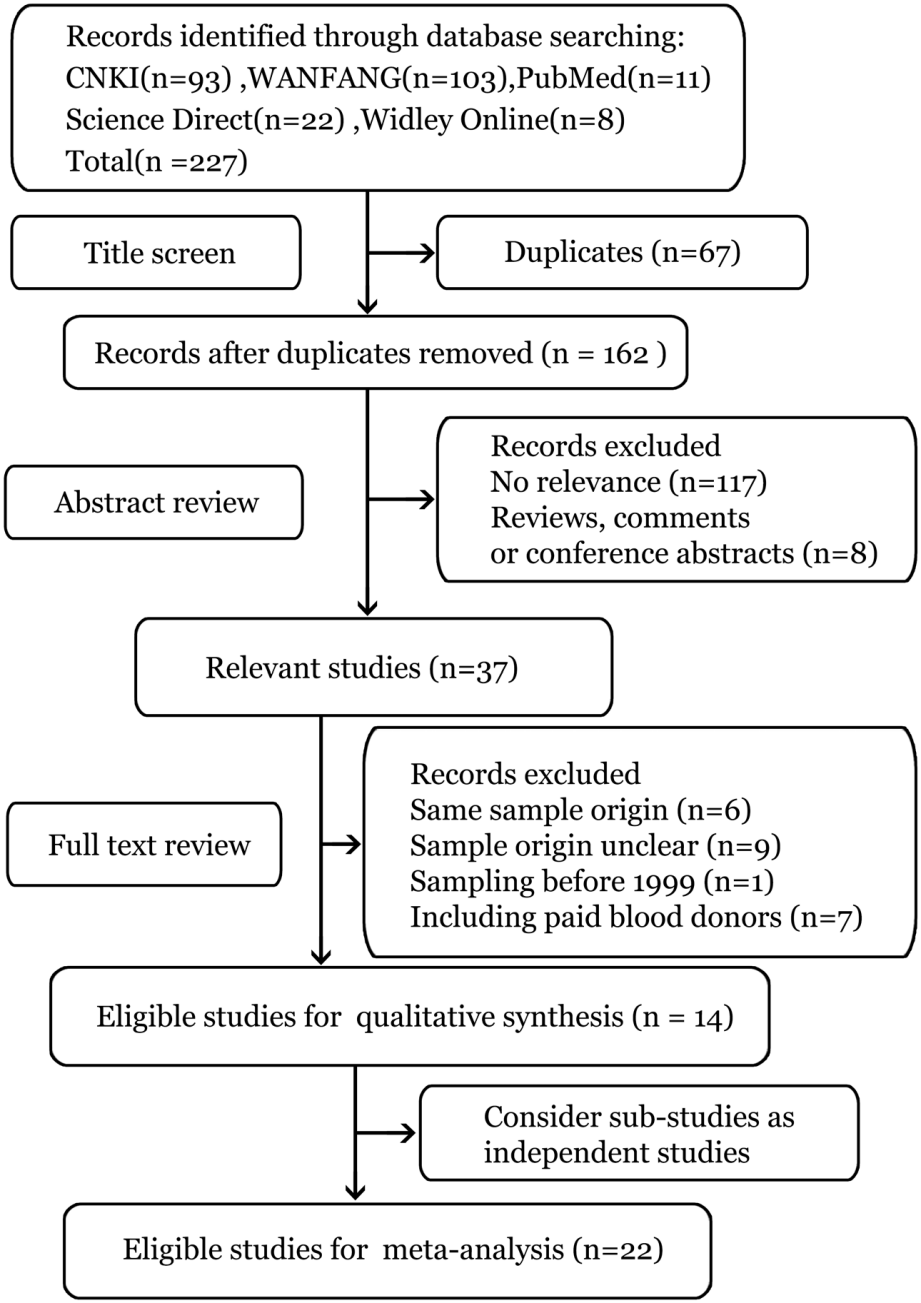
Flow chart of systematic literature search.

The initial search of the different combination of key terms identified a total of 227 records with 10 from PubMed, 20 from Science Direct, 8 from Widley Online, 80 from CNKI and 109 from Wanfang data. 67 duplicate records were removed by title screening. Then, the remaining 160 records were evaluated according to the inclusion and exclusion criteria, through which 123 studies were excluded by abstract screening and 16 studies were excluded by full text screening respectively. Finally, 14 records were selected for qualitative synthesis [7,18–30]. In the two studies with data from more than one region, the data in every region were considered as an independent study. Eventually, a total of 14 articles including 22 studies were obtained for the quantitative synthesis of the prevalence of HEV and 3 articles were selected for the analysis of HEV genotypes. The process of the study's selection was shown in Fig 1.

### Characteristics of the Selected Studies

All of the 14 selected papers were published in 2004 to 2014 and all the samples were collected from blood centers in 2001 to 2013. All of them were cross-sectional studies. In total, HEV serological prevalence estimates were derived from 100,470 participants from 18 cities of 9 provinces and autonomous districts and 1 municipality. The sample size of selected studies ranged from 163 to 20389 (median 3047, interquartile range 2250–4860). Among the studies, the information about both the gender ratio and the rate of samples with elevated ALT was available in 10 studies. The HEV infection biomarkers, IgG antibody, IgM antibody, antigen and RNA, were detected in 21, 21, 7 and 13 studies respectively. HEV serological and viremia prevalence was reported by these studies: from 11.66% to 41.71% for IgG, 0.43% to 2.80% for IgM, 0.02% to 0.20% for antigen and 0.02% to 0.20% for HEV RNA respectively. The details of every study were displayed in Table 1.

**Table 1:** Summary of data from eligible articles on HEV infection among voluntary blood donors in China.

Study	Location	Language	Year*	Gender ratio	Sample size	Case of elevated ALT	Serological reagents	Cases of IgG+	Cases of IgM+	Cases of RNA+	Cases of antigen+
Gao DY (2004)	Peking	Chinese	2002	1.8	7113	160	Wantai	1,891	124	NT	NT
Jia Y (2004)	Qinghai	Chinese	2002	0.73	4529	0	Wantai	1,161	NT	NT	NT
Cheng WG (2006)	Hubei	Chinese	2005	1.01	8213	NT	Wantai	NT	66	NT	NT
Wang HL (2005)	Liaoning	Chinese	2004	NA	163	1	Wantai	19	4	NT	NT
Sang LY (2007)	Zhejiang	Chinese	2005	1.39	3701	107	Wantai	1,107	50	6	NT
Liu XG (2008)	Hubei	Chinese	2006	NA	500	NT	Wantai	167	14	NT	1
Huang GY (2009)	Zhejiang	Chinese	2008	1.51	3044	45	Wantai	1013	28	3	NT
Wang XB (2009)	Liaoning	Chinese	2001	1.38	9376	0	Wantai	1687	85	NT	10
Guo QS (2010)	Zhejiang	English	2002	NA	3047	NT	Wantai	1271	46	6	NT
	Hubei	English	2005	NA	10136	NT	Wantai	3175	92	10	NT
	Fujiang	English	2005	1.3	20389	NT	Wantai	6215	186	4	NT
	Zhejiang	English	2006	1.83	4860	32	Wantai	1974	21	2	NT
	Zhejiang	English	2008	NA	2683	NT	Wantai	866	25	3	NT
Huang XY (2012)	Liaoning	Chinese	2010	NA	2250	407	Wantai	647	58	NT	0.5
Wang L (2013)	Zhejiang	Chinese	2012	1.72	4396	NT	Wantai	1788	43	NT	NT
Ren FR(2014)	Peking	English	2003	NA	2450	72	Wantai	474	32	NT	0.5
	Xinjiang	English	2003	NA	2157	247	Wantai	386	15	NT	1
	Yunnan	English	2003	NA	2272	319	Wantai	925	36	1	1
	Guangdong	English	2003	NA	2536	16	Wantai	751	17	1	2
	Zhejiang	English	2003	NA	2123	143	Wantai	728	31	1	4
Zhuang W(2014)	Jiangsu	English	2011	2.74	486	NT	Wantai	113	NT	NT	NT
Nie DM(2014)	Guangdong	Chinese	2013	1.9	4046	33	Wantai	896	50	NT	NT

*: the year of sampling started; if sampling time was not reported, it was computed by subtracting two of the year of publication (e.g., for an article published in 2010 with no sampling year reported, it is recorded as 2008;)

^+^: Positive

NA: not available

NT: not tested

### Pooled Prevalence of Every HEV Marker in Chinese Blood Donors

Pooled prevalence estimates of HEV infection markers (IgG antibody, IgM antibody, antigen and RNA) with corresponding confidence intervals (95%) were obtained from the analysis of all the 16 selected studies. a random effects model (DerSimonian and Laird method) was used to estimate the pooled prevalence of the three markers with anti-HEV IgG as 29.2% (95% CI 26.0–32.4), IgM as 1.1% (95% CI 1.0–1.3) and HEV RNA as 0.1%(95% CI 0–0.1), respectively as shown in Table 2. Meanwhile, pooled prevalence of HEV antigen was estimated at 0.1% (95% CI 0–0.1) by General Variance-Based model since no heterogeneity of it in the selected studies was observed (I^2^ antigen = 12.00%, P = 0.337).

**Table 2:** Pooled prevalence of HEV infection biomarkers.

HEV Infection Biomarkers	Number of Studies	Event Rate (%)	95% CI (%)	P Value	I^2^ Statistics
Anti-HEV IgG	21	29.2	26.0–32.4	0	99.10%
Anti-HEV IgM	20	1.1	1.0–1.3	0	84.40%
HEV RNA	10	0.1	0.00–0.1	0.031	47.7%
HEV antigen	8	0.1	0.00–0.1	0.337*	12.00%

*p>0.05

**Fig 2:**
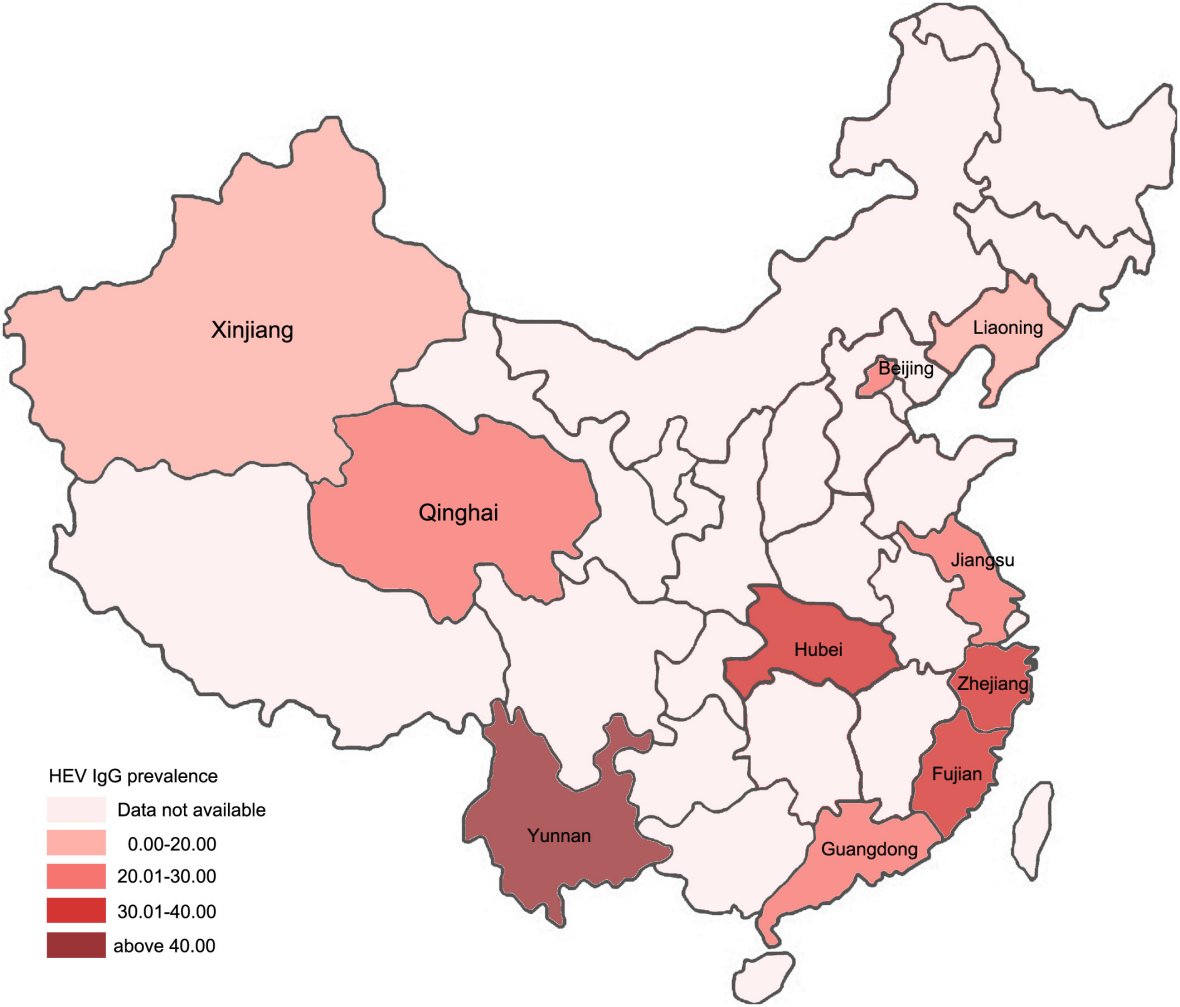
Geographical distribution of HEV IgG prevalence.

The moderators’ effect was investigated through the subgroup meta-analysis. Pooled prevalence of every marker of HEV in each subgroup was shown in Table 3. Through stratification by province, the highest and lowest pooled prevalence of anti-HEV IgG was estimated as 40.7% (95% CI 38.7–42.7) in Yunnan and 17.9%(95% CI 16.3–19.5) in Xinjiang respectively. Fig 2 showed the geographical distribution of HEV IgG prevalence. Heterogeneity of the prevalence of IgG shown by I^2^was substantial in the subgroups of provinces except for Hubei Province subgroup (I^2^ = 0%, p = 0.336). The pooled prevalence of anti-HEV IgM ranged from 0.4% (95% CI 0.1–0.7) to 1.9% (95% CI 0.4–3.3) in different province subgroups with substantial heterogeneity in all subgroups except for provinces with only one single study selected. The pooled prevalence of HEV RNA ranged from 0.00% to 0.4% (95% CI 0.1–0.7) in different subgroups of provinces. Low heterogeneity (I^2^ = 19.9%, P = 0.284) was observed in the subgroup of Zhejiang Province while the degrees of freedom were lower than 1 in other sub-groups. Through stratification by publication language, substantial heterogeneity of the prevalence of anti-HEV IgG and IgM was found in every subgroup.

**Table 3:** Meta-analysis of HEV infection indicators prevalence stratified by locations and languages.

Factors	HEV IgG					HEV IgM					HEV RNA					HEV antigen				
	N	Prevalence(%)	95%CI	P value	I^2^(%)	N	Prevalence(%)	95%CI	P value	I^2^(%)	N	Prevalence(%)	95%CI	P value	I^2^(%)	N	Prevalence(%)	95%CI	P value	I^2^(%)
*Location*																				
Peking	2	23	15.9–30.1	0	98.30	2	1.6	1.1–2.0	0.114*	59.90	0	NA				1	0	0.0–0.1		
Qinghai	1	25.6	24.4–26.9			0	NA				0	NA				NA				
Hubei	2	31.4	30.5–32.3	0.336*	0.00	3	0.9	0.6–1.3	0.024	73.20	1	0.1	0–0.2			1	0.2	(-0.2)-0.6		
Liaoning	3	19.7	11.2–28.2	0	98.30	3	1.9	0.4–3.3	0	91.80	0	NA				2	0.1	0.0–0.1	0.052*	73.50
Zhejiang	7	36.1	32.4–39.8	0	97.20	7	1.1	0.7–1.4	0	85.80	7	0.1	0.0–0.1	0.284*	19.90	1	0.2	0.0–0.4		
Fujian	1	30.5	29.9–31.1			1	0.9	0.8–1.0			1	0	0			NA				
Xinjiang	1	17.9	16.3–19.5			1	0.7	0.3–1.0			0	NA				1	0	0.0–0.1		
Yunnan	1	40.7	38.7–42.7			1	1.6	1.1–2.1			1	0	0.0–0.1			1	0	0.0–0.1		
Guangdong	2	25.9	18.5–33.2	0	97.80	2	1	0.4–1.5	0.017	82.60	1	0	0.0–0.1			1	0.1	0.0–0.1		
Jiangsu	1	23.3	19.5–27.0			0	NA				0	NA				NA				
*Language*																				
English	10	27.1	22.4–31.8	0	99.10	10	1.3	1.0–1.6	0	85.10	2	0.1	0–0.2	0.466*	0.00	3	0.1	0.0–0.1	0.118*	53.20
Chinese	10	31.1	27.1–35.1	0	98.90	10	1	0.8–1.2	0	81.80	8	0	0–0.1	0.094*	42.70	5	0.1	0.0–0.1	0.491*	0.00

*p>0.05

Furthermore, meta-regression was used to estimate the effect of study factors (sampling period, sample size, gender ratio, proportion of cases with elevated ALT) on the pooled prevalence of the HEV markers. The effect of the factors involving insufficient studies (less than 10) was not investigated to avoid the probability of type I error[31]. Details were given in Table 3. The proportion of cases with elevated ALT (adjusted R^2^ = 29.55%, p = 0.058) was the sole contributor to the heterogeneity of the prevalence of anti-HEV IgM. Studies with higher proportion of ALT elevated cases tend to report a higher prevalence of anti-HEV IgM. Sample size (adjust R^2^ = 61.65%, p = 0.89) but not the sampling year (p = 0.758) or gender ratio (p = 0.920) contributed significantly to the heterogeneity of the prevalence of HEV RNA. None of the four factors contributed significantly to the heterogeneity of the prevalence of anti-HEV IgG.

The Publication bias was assessed by Egger’s test[32]. No significant publication bias was found for the prevalence of anti-HEV IgG (p = 0.423), HEV RNA (p = 0.904) and HEV antigen (P = 0.874). However, statistically significant publication bias (P = 0.003) was observed for the prevalence of HEV IgM.

### Genotypes of HEV among Infected Blood Donors

Among the 14 selected articles, 3 articles reported the genotypes of HEV [22,24,29]. HEV RNA was sequenced directly on both strands with specific primers and then the sequence was aligned with the sequence of the standard strains of the four HEV genotypes. Phylogenetic analyses indicated that HEV isolated from the donor samples were closely related to genotype 1 and 4, (genotype 4 in Yunnan and Guangzhou, genotype 1 and 4 in Zhejiang). For the only one article reported the HEV subtypes, the HEV isolates from Yunnan, Guangzhou and Zhejiang showed the highest identities to HEV subtypes 4a, 4b and 4d, respectively.

## Discussion

The blood donors have not been screened for HEV routinely yet in any country. However, considerable studies focusing on HEV infection among blood donors have been conducted in many countries and regions to evaluate the risk of HEV infection transmitted by transfusion[33]. The prevalence of anti-HEV IgG in blood donors was reported as 4.9% to 52.5% (Midi-Pyrenees, France) in Europe, 5.3% in Australia, 14.3% to 21.48% in center Asia, and 22.3% in the United States [34–41]. The prevalence of anti-HEV IgM in blood donors was investigated as 4.78% and 4.3% in India and Saudi Arabia, respectively[40,41]. The prevalence of HEV RNA in blood donors was considered to be 0.045% in French, 0.031% in Dutch, 0.03% in Spain and 0.08% in Germany[34,36,42]. Among which, the prevalence of anti-HEV IgG among blood donors had always been reported while the prevalence of other markers was not reported so frequently. To have a comprehensive look of the prevalence of HEV in Chinese blood donors, we estimated the pooled prevalence with all the available markers of HEV including anti-HEV IgG, IgM, HEV RNA and antigen. Pooled prevalence of anti-HEV IgG, IgM, HEV RNA and antigen of blood donors in China was found to be 29.2%, 1.1%, 0.1% and 0.1%, respectively and appeared to be much higher, especially for HEV RNA positive rate, than that in most other regions. Meanwhile, differences between IgG prevalence were observed across provinces in China. Similar to other studies, our findings showed a higher anti-HEV IgG prevalence among provinces located in southern China which may suggest that geographical region is an important factor for HEV infection[29]. In Liaoning province, IgM prevalence was found to be higher than that of other regions (p = 0.311), since one of the studies performed in Liaoning collected samples from a garrison army in which 2 soldiers were diagnosed as hepatitis E[21]. Similar with the report in France in 2011[37], no evidence of a relation between gender and prevalence of HEV infection biomarkers was observed in our study while other studies indicated higher anti-HEV prevalence among males[29]. Moreover, we didn’t find trends of HEV biomarker prevalence in blood donors changing with sampling year. It seemed that the proportion of donors with elevated ALT contributed to the discrepancy of the prevalence of anti-HEV IgM and antigen. The prevalence of IgM tended to be higher with the higher proportion of donors with elevated ALT. However, no obvious correlation was observed between the prevalence of HEV antigen and the proportion of donors with elevated ALT which may result from that ALT usually elevated after the specific immune response with the elimination of antigen. It was ever reported in France that most samples (22/24) from viremic donors were negative for IgG and IgM against HEV in the screening of 53,234 blood donors[42]. RNA detection in the studies including in our meta-analysis was performed only for anti-HEV IgM or antigen positive donors, which might underestimate the prevalence of HEV RNA in our study. Under this circumstance, the equally pooled prevalence of HEV antigen and RNA in our study can suggest that the anti-HEV IgG and IgM testing may not help to eliminate HEV infectious blood donation because IgG is a marker of previous infection and IgM disappears early in the convalescent period which may further propose HEV RNA detection as “golden standard” for detecting HEV infectious blood donors[43].

Molecular epidemiology study of HEV strains among humans showed different geographical distributions of different genotypes with genotype 1 prevalent in Asia and North Africa, genotype 2 in Mexico and Southern Africa, genotype 3 in North and South America, Europe and Asia) and genotype 4 in Asia. The strains of genotype 1 and 2 are restricted to humans, whereas genotype 3 and 4 are usually zoonotic. In China, the prevalent genotypes of HEV are genotype 1, 3 and 4 in general population[44]. According to our analysis, the strains of genotype 1 and 4 are more prevalent than genotype 3 in Chinese blood donors.

This meta-analysis does have multiple limitations: 1) not all of the 31 provinces in mainland China were listed since the data were not available in some regions.; 2) the demographic information were insufficient, with the mean age as an example, since these data were not reported in some of the selected studies; 3) bias and confounding results appeared to be inevitable given that the sampling methods of some selected studies were not quite clear.

## Conclusion

In summary, hepatitis E is one of the old diseases with new implications which may be a threat to the safety of blood and blood products. Our study demonstrated the seroprevalence and viremic prevalence of HEV among blood donors in partial areas in China and found that the prevalence of HEV infection was relatively higher than that in other countries. Since not all the samples were screened for HEV RNA in the studies included in the analysis, the prevalence of HEV RNA might be underestimated in our study, thus, the real HEV RNA prevalence might be higher in Chinese blood donors. Therefore, China is indicated with a relatively higher risk of transmission of hepatitis E through transfusion and the screening of blood donors for HEV RNA, especially in HEV-endemic areas, might reduce the potential risk of HEV infection via transfusion.

## Supporting Information

S1 Fig Forest Plots of HEV IgG Estemates. (TIF)

S2 Fig Forest Plots of HEV IgM Estemates. (TIF)

S3 Fig Forest Plots of HEV RNA Estemates. (TIF)

S4 Fig Forest Plots of HEV Antigen Estemates. (TIF)

S1 Table PRISMA Checklist. (DOC)

S2 Table Search strategy. (DOCX)
