# Supplementary figures and images for: Acute, Recent and Past HEV Infection among Voluntary Blood Donors in China: A Systematic Review and Meta-Analysis
Source: PLoS One. 2016 Sep 6;11(9):e0161089. doi: 10.1371/journal.pone.0161089 (PMC5012590; doi:10.1371/journal.pone.0161089)

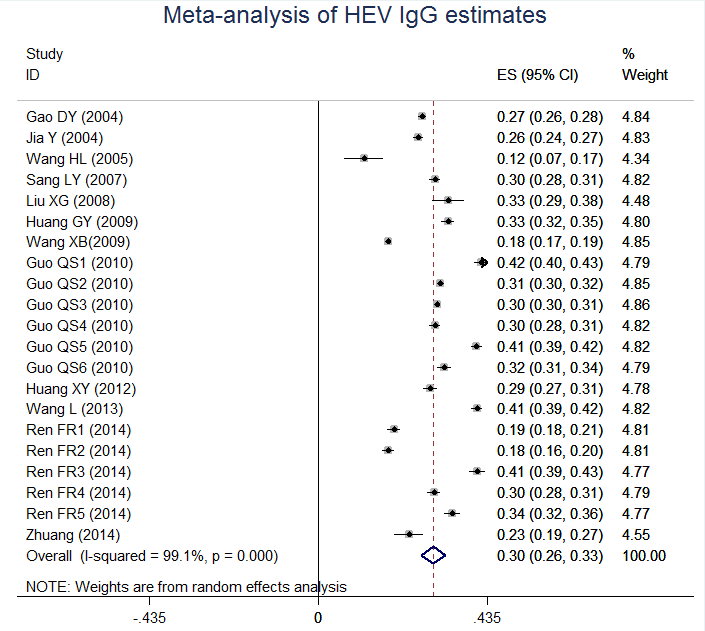

Supplement: S1 Fig — (TIF) [file pone.0161089.s001.tif]

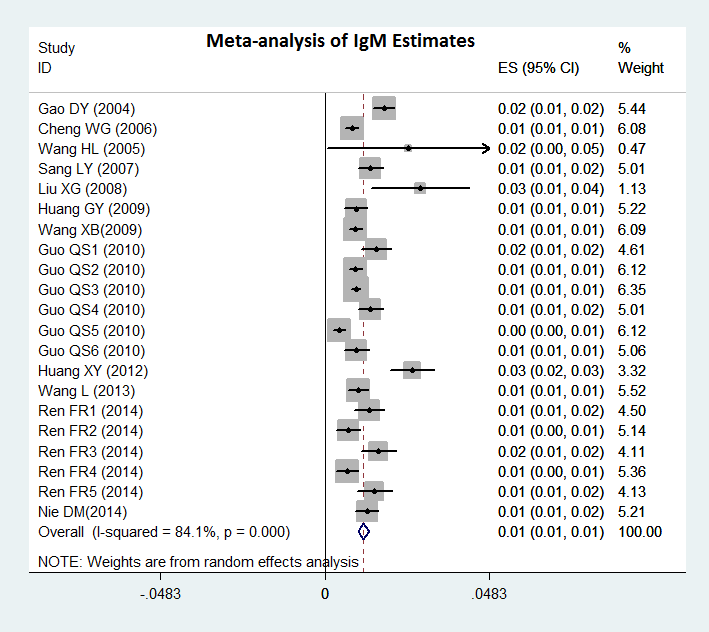

Supplement: S2 Fig — (TIF) [file pone.0161089.s002.tif]

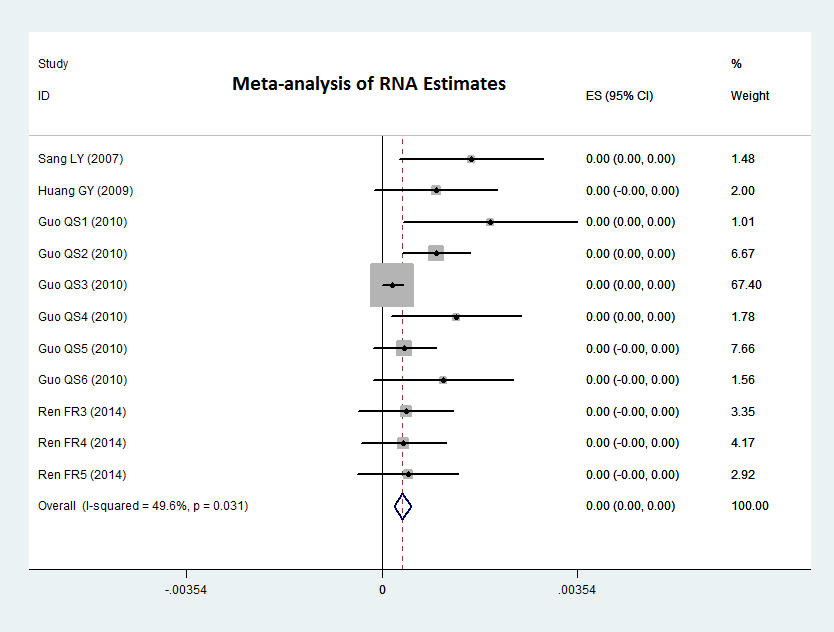

Supplement: S3 Fig — (TIF) [file pone.0161089.s003.tif]

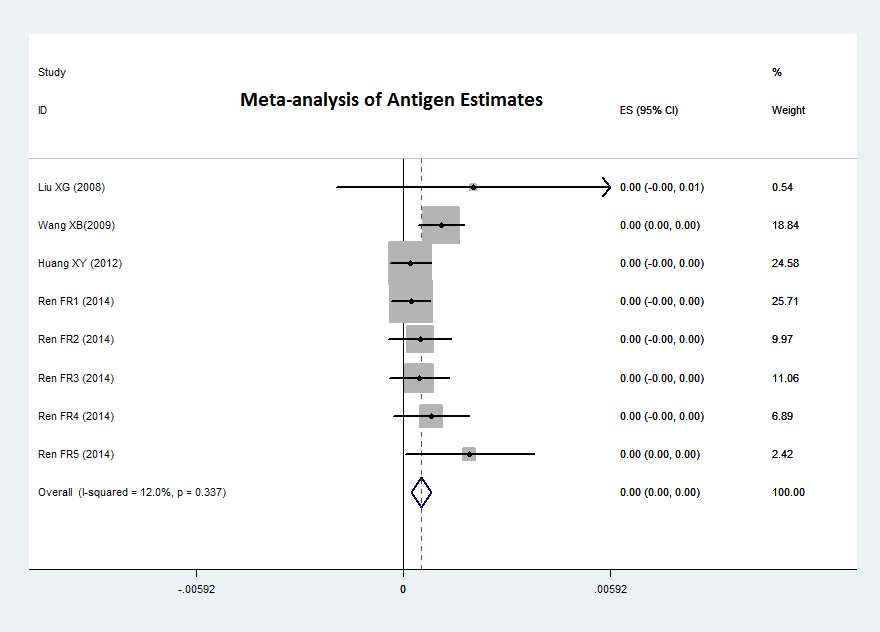

Supplement: S4 Fig — (TIF) [file pone.0161089.s004.tif]
