# Supplementary material for: Acute, Recent and Past HEV Infection among Voluntary Blood Donors in China: A Systematic Review and Meta-Analysis
Source: PLoS One. 2016 Sep 6;11(9):e0161089. doi: 10.1371/journal.pone.0161089 (PMC5012590; doi:10.1371/journal.pone.0161089)
Supplement: S2 Table — (DOCX) [file pone.0161089.s006.docx]

| **Data Base** | **Method** | **Search Strategy** | **Number of Results** |
| --- | --- | --- | --- |
| PubMed | Advanced search | (hepatitis e[Title/Abstract] OR HEV[Title/Abstract]) AND (blood donors [Title/Abstract] OR donation[Title/Abstract]) AND (china[Title/Abstract] OR Chinese[Title/Abstract]) | 10 |
| Scince Direct | Advanced search | ("hepatitis e" OR hev) AND ("blood donors" OR donation) AND abs (China or Chinese) | 21 |
| Wiley online | Advanced search | HEV in Abstract AND “blood donors” in Abstract AND *CHINESE in FullText NOT TAIWAN in Abstract | 8 |
